# Application of pectin hydrolyzing bacteria in tobacco to improve flue-cured tobacco quality

**DOI:** 10.3389/fbioe.2024.1340160

**Published:** 2024-03-07

**Authors:** Shuning Weng, Meizhong Deng, Shanyi Chen, Renqiang Yang, Jingjing Li, Xianbo Zhao, Shunhua Ji, Lixiang Wu, Li Ni, Enren Zhang, Chaochao Wang, Lingfeng Qi, Kuanqi Liao, Yiqiang Chen, Wen Zhang

**Affiliations:** ^1^ Institute of Food Science and Technology, College of Biological Science and Engineering, Fuzhou University, Fuzhou, Fujian, China; ^2^ Technology Center, China Tobacco Fujian Industrial Co., Ltd., Xiamen, Fujian, China; ^3^ Xiamen Tobacco Industrial Co., Ltd., Xiamen, Fujian, China

**Keywords:** flue-cured tobacco, microorganism, pectin, quality, flavor

## Abstract

To study the relationship between the diversity of the surface microbial community and tobacco flavor, and to improve tobacco quality using microorganisms. The microbial community composition and diversity of 14 samples of flue-cured tobacco from tobacco-producing areas in Yunnan with varying grades were analyzed by high-throughput sequencing. PICRUSt was used for predicting microbial functions. A strain of *Bacillus amyloliquefaciens* W6-2 with the ability to degrade pectin was screened from the surface of flued-cured tobacco leaves from Yunnan reroasted tobacco leave. The enzyme preparation was prepared through fermentation and then applied for treating flue-cured tobacco. The improvement effect was evaluated by measuring the content of macromolecule and the changes in volatile components, combined with sensory evaluations. The bacterial communities on the surface of flue-cured tobacco exhibited functional diversity, consisting primarily of *Variovorax*, *Pseudomonas*, *Sphingomonas*, *Burkholderia*, and *Bacillus*. These bacterial strains played a role in the aging process of flue-cured tobacco leaves by participating in amino acid metabolism and carbohydrate metabolism. These metabolic activity converted complex macromolecules into smaller molecular compounds, ultimately influence the smoking quality and burning characteristics of flue-cured tobacco. The pectinase preparation produced through fermentation using W6-2 has been found to enhance the aroma and sweetness of flue-cured tobacco, leading to improved aroma, reduced impurities, and enhanced smoothness. Additionally, the levels of pectin, cellulose, and hemicellulose decreased, while the levels of water-soluble sugar and reducing sugar increased, and the contents of esters, ketones, and aldehydes increased, and the contents of benzoic acid decreased. The study revealed the correlation between surface microorganisms and volatile components of Yunnan tobacco leaves, and the enzyme produced by the pectin-degrading bacteria W6-2 effectively improved the quality of flue-cured tobacco.

## 1 Introduction

Tobacco undergoes several processes from growth in the field to becoming a cigarette product, which include primary roasting, cleaning, re-roasting, storage, aging, silk-making, and packet rolling. Roasted tobacco that has not undergone aging is unsuitable for processing into cigarette products as it produces pungent and unpleasant odors, unpleasant aromas, and irritates smokers (Wang et al., 2015). Pectin, which is a polymer of galacturonic acid formed by α-1,4 glycosidic bonds, is an important component in tobacco. Pectin has a hydrophilic colloid structure that can maintain the moisture and toughness of tobacco leaves. However, an excessive amount of pectin in tobacco can lead to incomplete combustion of cigarettes, and the decomposition of pectin into small molecules such as methanol (Dorokhov et al., 2015) can result in a decrease in the taste and quality of tobacco. In addition, pectin can converted into acetic acid during the aging process of tobacco leaves, causing coughing, throat irritation, and a pungent and irritating odor. When it is burned and decomposed, it can also produce volatile harmful substances, which are harmful to the health of smokers (Wang et al., 2013). Cellulose, as the basic substance of tobacco cell tissue and skeleton, is the main component of tobacco cell wall (Shen and Gu, 2009). About 11% of tobacco leaves are cellulose, and too much cellulose in tobacco leaves can produce pungent smoke that makes smokers bitter. Torikaiu et al. (Torikaiu et al., 2005) added microcrystalline cellulose powder to tobacco to simulate the cellulose pyrolysis model in tobacco leaves, and the results showed that the formaldehyde content of the pyrolysis products increased significantly, further indicating that the cellulose content was negatively correlated with the quality of tobacco leaves, and even caused health problems.

Microorganisms play an essential role in the aging of tobacco (Reid et al., 1937). Along with the time with natural fermentation, the microbes will form dominant microbial populations, which respond to the main affection of fermentation with tobacco leaves. Furthermore, in the period of late fermentation, bacteria become dominant strains compared to fungi (Zhou et al., 2020), and display obvious biodiversity (Hu et al., 2021). The use of functional strains for biofortification is an effective way to reduce the aging time and improve the organoleptic quality of smoked tobacco. It was found that potential synergy exists among microbes and enzymes by metagenomics analysis of fermented tobacco leaves (Tao et al., 2022). During the metabolic process, enzymes secreted by microorganisms that are on the surface of tobacco leaves can convert large molecules, such as starch, protein, pectin, cellulose, and lignin, into small molecules. The use of enzymes produced by microorganisms to degrade large molecules is crucial for improving the quality of tobacco leaves, reducing aging time, and increasing production and economic efficiency. Yu et al. (2009) treated tobacco leaves with the addition of exogenous pectinase, which reduced the pectin content in tobacco leaves, significantly increased the contents of geranyl acetone, macrobean trienone and neophytadiene, and the total amount of aroma substances increased by 29.94%. Deng et al. (2003) isolated the pectinase-producing strain DPE-005 from Yuxi flue-cured tobacco in Yunnan and sprayed the fermentation solution on flue-cured tobacco, which effectively reduced the pectin content and cell wall substance content of the tobacco. This also reduced the irritation and stray gas produced during smoking, improving the value of the tobacco. Zhang et al. (2024) screened *Bacillus velezensis* A2 and *Bacillus endophyticus* A4 from tobacco leaves. Co-fermentation of the two strains could effectively reduce the contents of starch, cellulose and protein in tobacco, resulting in an increase in reducing sugar content. The contents of solanone, methyl palmitate and dihydrokiwi lactone were significantly increased, and the total aroma component content was increased by 108.33%, which significantly improved the sensory quality of cigarettes. Wu et al. (2021) screened *Bacillus amylolyticus* LB and *Bacillus licheniformis* SC for α-amylase production using a protease from flue-cured tobacco. The researchers co-cultured the two strains and observed an increase in the levels of most Maillard reaction products and terpene metabolites in the tobacco. This led to an increase in aroma and a decrease in softness and irritation.

Aroma component is the basis of aroma quality of tobacco leaves, and aroma component is the basis of aroma quality of tobacco leaves, generally divided into acidic, alkaline and neutral aroma components. Among them, neutral aroma components are important components of tobacco aroma, mainly composed of ketones, formaldehyde, olefins, esters and alcohols, which make cigarettes sweet, fragrant and fruity, and make the smoke more mellow (Dai, 2013). Acidic aromas are mainly lower fatty acids (below C12), while alkaline aromas are usually nitrogenous compounds, including pyrrole, pyridine and pyrazine compounds that have important odorant effects (Longjie et al., 2020). Wu et al. (1992) found that neutral volatile substances, including neophytadiene, glycoaldehyde, furfuryl alcohol and damarone, had the greatest influence on the flavor quality of tobacco leaves, which could be used as one of the criteria for judging the aroma quality of tobacco leaves. Therefore, exploring the changes of volatile components in tobacco leaves is one of the important means to predict the improvement of tobacco quality.

This study focuses on the surface microbiota of Yunnan tobacco leaves after re-roasting, and uses a combination of molecular biology and bioinformatics to reveal the contribution of bacterial diversity in the tobacco microbiota to the quality of tobacco, as well as the pathways involved in regulating quality. Furthermore, beneficial bacterial strains that can break down pectin in tobacco are selected and processed into enzyme preparations, which are then applied onto tobacco leaves. This process quickly and specifically improves the quality of tobacco by enhancing the coordination of chemical components, increasing the concentration of aromatic substances, reducing the levels of impurities and irritants in smoke, and effectively enhancing the quality of flue-cured tobacco.

## 2 Materials and methods

### 2.1 Materials

#### 2.1.1 Tobacco samples

The samples used in this experiment were selected from Yunnan tobacco after being reroasted and provided by the Technical Center of Fujian China Tobacco Industry Co., Ltd (Table 1).

**TABLE 1:** Samples of tobacco leaves of different grades in Yunnan.

Number	Source/year/grade
Y1	Yunnan MaZhan YLC1Y-2019 piece of tobacco (not rated)
Y2	Yunnan MaZhan C4FY-2018 piece of tobacco (not rated)
Y3	Yunnan C-2019 Terrier (not rated)
Y4	Yunnan Pu’er Jingdong ESLC1Y-2019 Flake tobacco (high grade)
Y5	Yunnan Chuxiong YLC1-2019 piece of tobacco (high grade)
Y6	Yunnan Dali Xiangyun YLC1Y-2019 piece of tobacco (high grade)
Y7	Yunnan MaZhan ELC1Y-2019 piece of tobacco (medium tobacco)
Y8	Yunnan Chuxiong ELC1-2019 piece of tobacco (medium tobacco)
Y9	Yunnan Dali Xiangyun ELC1Y-2019 piece of tobacco (medium tobacco)
Y10	Yunnan Quchu Da F03-2019 piece of tobacco (low grade)
Y11	Yunnan Chuxiong F03-2019 piece of tobacco (low grade)
Y12	Yunnan Tobacco Company B2F-2019 Flake tobacco (not rated)
Y13	Yunnan Tobacco Company C3F-2019 Flake tobacco (not rated)
Y14	Yunnan Ma Zhanda X2F-2019 flake tobacco (not rated)

#### 2.1.2 Strain and medium

*Bacillus amyloliquefaciens* W6-2 was isolated from Yunnan tobacco leaves samples by the Institute of Food Science and Technology at Fuzhou University and identified using the 16S rDNA method by Bioengineering (Shanghai) Co., LTD. (Accession No. OQ979111).

TSB medium: tryptone 17.0 g, soybean peptone 3.0 g, sodium chloride 5.0 g, dipotassium hydrogen phosphate 2.5 g, glucose 2.5 g, distilled water 1,000 mL, pH = 7.3 ± 0.2.

Optimized medium for pectinase production: pectin 5 g, xylose 5 g, bacteriological peptone 6 g, ZnSO_4_·7H_2_O 5 g, distilled water 1,000 mL, pH natural.

Optimized medium for cellulase production: sodium carboxymethyl cellulose 10 g, yeast powder 10 g, KH_2_PO_4_ 1 g, distilled water 1,000 mL, pH natural.

### 2.2 Microbial enrichment, high-throughput sequencing and functional prediction of tobacco surface

Fully soak 10 g of tobacco leaves with 60 mL 0.01 mol/L PBS solution (pH 7.4), shaked the shaking table at 180 rpm per min for 40 min, and used 0.22 μM filter the solution with a membrane, and then eluted the bacteria adhering to the membrane with 40 mL PBS.

The bacterial samples were subjected to high-throughput sequencing using the Illumina MiSeq sequencing platform (Shanghai Meiji Bio-pharm Technology Co., Ltd.). The DNA of microorganisms on the surface of Yunnan tobacco leaves was extracted according to the steps of environmental genomic DNA extraction kit. After 1% agarose gel electrophoresis was performed to detect the quality of extracted gene DNA, PCR was used to amplify the V3-V4 variable region of 16S rDNA of bacteria using citation 338F (ACT​CCT​ACG​GGA​GGC​AGC​AG) and 806R (GGACTACHVGGGTWTCTAAT). After mixing according to the sequencing amount of each sample, High-throughput sequencing of amplicon was performed on Illumina platform.

The high-throughput data analysis was performed using the Majorbio Cloud Platform. The potential functional composition of the microbial community was predicted using the PICRUSt software package, and the correlation between flavor and strains was analyzed using Cytoscape software.

### 2.3 Production and enzyme activity determination of crude pectinase preparation

*Bacillus amyloliquefaciens* W6-2 was activated and inoculated into 100 mL of TSB liquid medium, followed by cultivation at 30°C and 200 rpm for 12 h. The activated culture was then transferred into a 200 L fermenter at a ratio of 1:100 and fermented for 24 h at 30°C and 130 rpm. After fermentation, the liquid was subjected to sterilization using a 200 nm ceramic membrane, followed by filtration through a 100 kDa ultrafiltration membrane. Finally, the liquid was concentrated using a 30 kDa ultrafiltration membrane to obtain the enzyme preparation.

The activity of pectinase was determined by the Gross method (Ben-Arie and Sonego, 1980) with slight modifications. A reaction mixture containing 1.8 mL of pectin solution and 0.2 mL of fermentation broth was incubated at 50°C for 30 min. Subsequently, 3.0 mL of DNS reagent was added and boiled in a water bath for 10 min. Finally, the reaction was cooled to room temperature, adjusted to a constant volume, and a UV-Vis spectrophotometer was used to measure the absorbance of a galacturonic acid standard curve at 540 nm. The method quantifies galacturonic acid, which was the reaction product that was catalyzed. One unit of activity was defined as the amount that could decompose pectin to produce 1 μmol galacturonic acid (U/mL) per min.

Cellulase activity was determined using the Bunti V method (Buntić et al., 2019) with a slight modification: a reaction mixture containing 1 mL of sodium carboxymethyl cellulose solution and 1 mL of fermentation broth was incubated at 50°C for 30 min. Subsequently, 2.5 mL of DNS reagent was added and boiled in a water bath for 10 min. Finally, the reaction was cooled to room temperature, adjusted to a constant volume, and a UV-Vis spectrophotometer was used to measure the absorbance of a glucose standard curve at 550 nm. The cellulase activity of one unit was defined as the amount required to produce 1 μg of glucose per min.

### 2.4 Sensory evaluation

Roasted tobacco leaves (B03-2020, Nanping, Fujian) that have been processed into cut tobacco were selected as samples for sensory testing. To simulate the operation process in the workshop, approximately 50 g of tobacco leaves were taken and spread onto a sample tray. According to the required treatment concentration at a ratio of 5% (w/w), a pectinase preparation was evenly sprayed onto the tobacco leaves using a spray. After being left at room temperature for 6 h, the enzyme was deactivated at 135°C for 1 min. The treated samples were then placed in bags and kept in a constant temperature and humidity incubator for 48 h to allow for moisture balance at 22°C and a relative humidity of (60 ± 5)%. For comparison, control samples were made using the same amount of water and inactivated enzyme liquid. The sample rolls obtained were made into cigarettes and randomly numbered. Sensory evaluation of the cigarettes was conducted by at least 7 sensory evaluation experts following the standard GB 5606.4-2005 Cigarette Part 4: Sensory Technology.

### 2.5 Determination of conventional chemical components of tobacco leaves

Both water-soluble total sugar and reducing sugar were determined using the continuous flow method, following the tobacco industry standard YC/T 159-2002.

The contents of starch, protein, pectin, cellulose, hemicellulose and lignin were determined according to the tobacco industry standards YC/T 216-2013, YC/T 249-2008, YC/T 346-2010 and YC/T 347-2010, respectively.

### 2.6 Determination of volatile components in tobacco leaves

Volatile compounds were analyzed using headspace solid-phase microextraction coupled with gas chromatography-mass spectrometry (HS-SPME-GC-MS). Volatile head space compounds were collected in a 50:30 μm SPME fiber (DVB/CAR/PDMS). Prior to use, the fiber was aged for 10 min at 250°C in the gas chromatography injection port. In a 15 mL screw-cap headspace vial, 0.4 g of tobacco leaves and 3 μL of 3.432 mg/L phenethyl acetate were added as an internal standard. The extraction fiber was inserted into the headspace vial and preheated for 10 min at 50°C before being released to extract the volatile compounds for 30 min. Finally, the volatile compounds were analyzed for 3 min at 250°C in the GC-MS injection port.

The chromatographic column was HP-5MS column (60 m × 0.25 mm×0.25 μm) without shunt injection. Helium (99.999% purity) was used as carrier gas at a constant flow rate of 1 mL/min. According to the method of Wang et al. (Wang et al., 2008), the heating procedure of the chromatography was as follows: the initial temperature was 50°C, kept for 2 min, and then the temperature was heated at 8°C/min to 280°C, and kept for 25 min. After operation temperature 280°C, after operation time 5 min. Solvent delay 4 min. The mass spectrum conditions was as follows: EI mode of 70 eV, ion source temperature, 230°C. The MS analysis was carried out in full-scan mode, with a scan range of m/z 35–350.

NIST 14 standard spectrum library was used to identify the substances and conduct qualitative analysis. The quantitative method is calculated by internal standard method.

### 2.7 Statistical analysis

All samples were conducted at least in triplicate and the results were expressed as means ± standard deviation. Statistical analysis of the data was performed using Statistical Package for the Social Sciences (SPSS) 23.0 (IBM, Armonk, New York). Student’s t-test was used for two-group comparisons (*p < 0.05, **p < 0.01).

## 3 Results

### 3.1 Prediction of the function of bacterial communities on the surface of tobacco leaves

#### 3.1.1 Bacterial diversity on the surface of flue-cured tobacco

**FIGURE 1:**
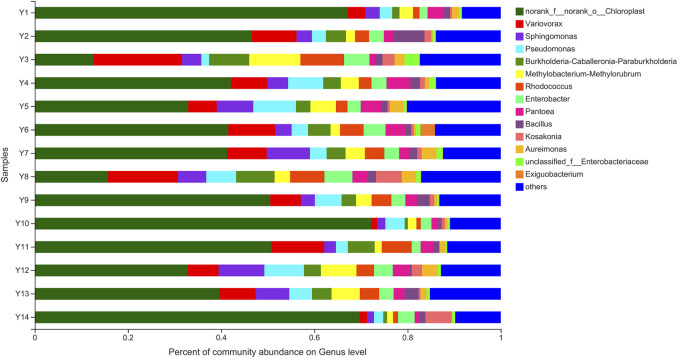
Histogram of bacterial colony structure at genus level.

At the level of genus, genera with a relative abundance of less than 1% are categorized as “Other”. Figure 1 shows the top 10 bacteria genera in 14 samples of tobacco leaves from Yunnan in terms of relative abundance: *Variovorax*, *Sphingomonas*, *Pseudomonas*, *Burkholderia-Caballeronia-Paraburkholderia*, *Methylobacterium-Methylorubrum*, *Rhodococcus*, *Enterobacter*, *Pantoea*, *Bacillus*, and *Kosakonia*. *Bacillus*, a genus of spore-forming bacteria, can promote tobacco fermentation and the formation of flavor compounds (English et al., 1967). It is also the most reported enzyme-producing dominant bacteria. For example, *Bacillus subtilis* can accelerate the formation of desirable flavors and improve the smoking quality of cigar smoking (Kaelin and Gadani, 2000). Zhou et al. (Man et al., 2017) isolated pectin-degrading bacteria from tobacco leaves and identified *Bacillus subtilis* PB1 as the most active bacterium through identification of the 16S rRNA. By optimizing the production process of pectinase using response surface methodology (RSM), they effectively reduced the content of pectin in tobacco leaves.

**FIGURE 2:**
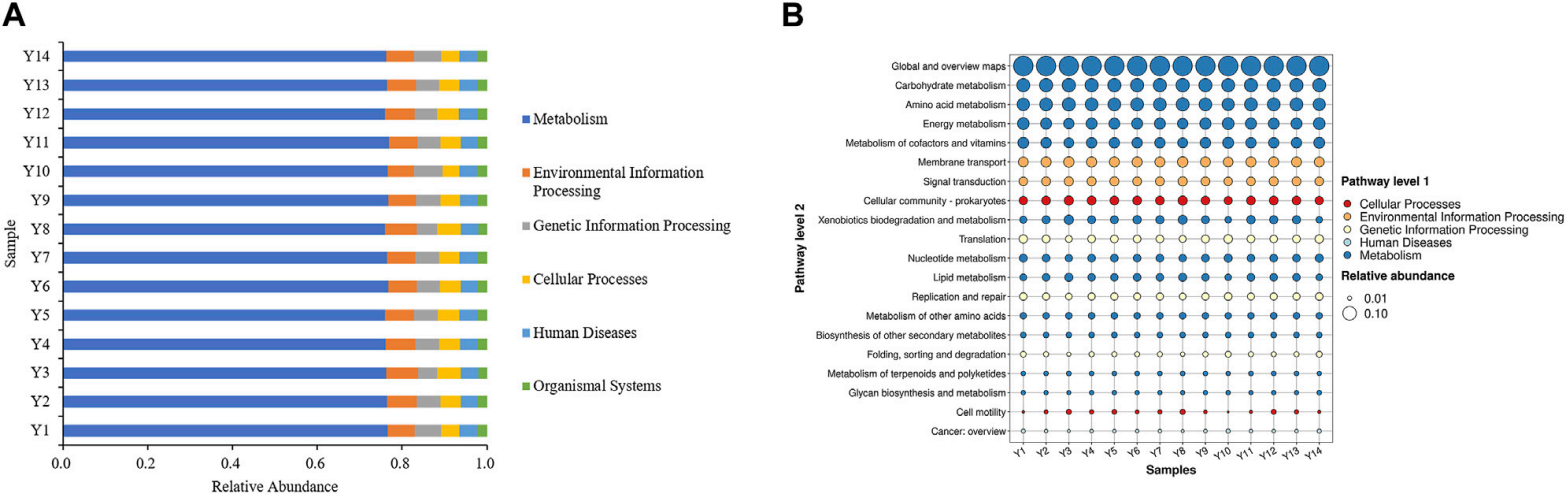
**(A)** Primary functional analysis of predicted genes; **(B)** Secondary function analysis of predictive genes.

The bacterial community sequencing data from the 14 Yunnan tobacco samples was analyzed using PICRUSt prediction, which is a tool used to predict the potential functions of microbial communities in samples (Shiratori et al., 2009). The results are presented in Figure 2A. The prediction results based on the KEGG database showed that the KEGG pathway richness of all the samples was similar, and all involved six types of biometabolic pathways in the primary functional layer. Metabolism had the highest percentage in each sample, exceeding 75% indicating that microorganisms on the surface of flue-cured tobacco participate in the aging process of flue-cured tobacco mainly through metabolic pathways.

The secondary functional layer of the predicted genes was analyzed in further detail, encompassing 46 secondary and 400 tertiary sub-functions. Figure 2B presents the top 20 sub-functional layers, along with their respective abundance in the secondary functional layer. The results indicated that the top 20 sub-functions accounted for over 90% of the total samples. Among these, the metabolic function of the primary functional layer accounted for 60%. Based on these findings, it is speculated that the microorganisms present in Yunnan flue-cured tobacco primarily participate in the transformation of chemical components in tobacco through activities such as Carbon metabolism and Biosynthesis of amino acids. These activities contribute to the formation of aroma precursors, which play a crucial role in improving the smoking quality of cigarettes and enhancing the overall quality of tobacco.

Based on the analysis of bacterial community function, carbon metabolism was the main pathway through which microorganisms in Yunnan flue-cured tobacco participate in the transformation of chemical components in tobacco. Therefore, screening for dominant strains that can specifically degrade macromolecules in tobacco has a positive effect on improving tobacco flavor.

**FIGURE 3:**
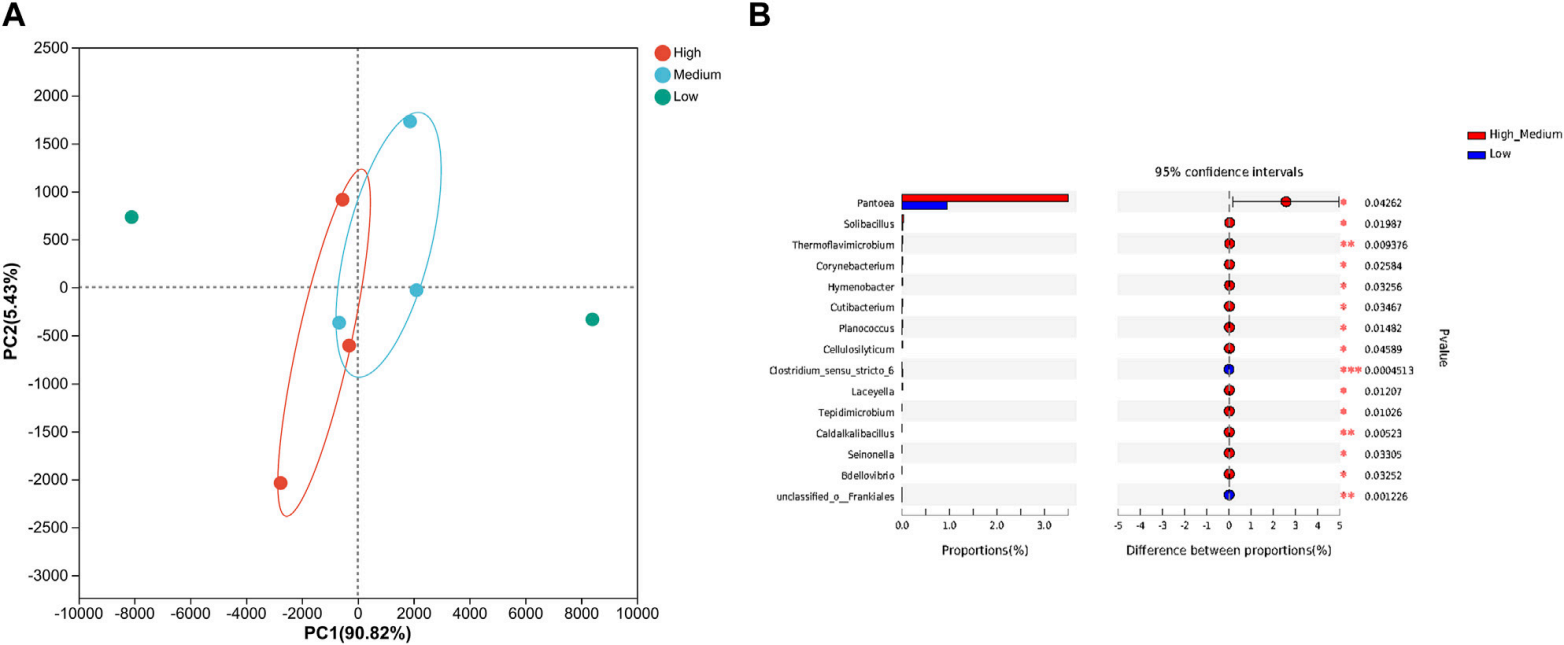
Principal component analysis and difference analysis of bacteria with different quality grades. **(A)** Principal component analysis; **(B)** difference analysis. L: low grade tobacco; M: medium grade tobacco; H: High grade tobacco.

The PCA analysis revealed differences in microbial community composition among the 8 Yunnan tobacco samples with different grades. As shown in Figure 3A, there were minimal variations in bacterial community at the genus level between the high-graded and medium-graded tobacco samples in Yunnan. However, both the high and medium-graded tobacco samples exhibited significant differences in bacterial community composition compared to the low-graded tobacco samples.

The results of Welch’s *t*-test are shown in Figure 3B. The main differential bacteria between high, medium, and low-graded tobacco in Yunnan were *Pantoea*, *Solibacillus*, *Cellulosilyticum*, *Clostridium sensu stricto*, and *Tepidimicrobium*, etc. The dominant bacteria were all found in the high and medium grades of tobacco in Yunnan, and most of them have macromolecular degradation abilities. For example, *Solibacillus* is capable of producing amylase, lipase, and protease (Cheng et al., 2020). *Cellulosilyticum* and *Clostridium sensu stricto* are cellulose-degrading bacteria (Pan et al., 2021). *Tepidimicrobium* can break down polysaccharides, proteins, and other substances (Dai et al., 2017).

**FIGURE 4:**
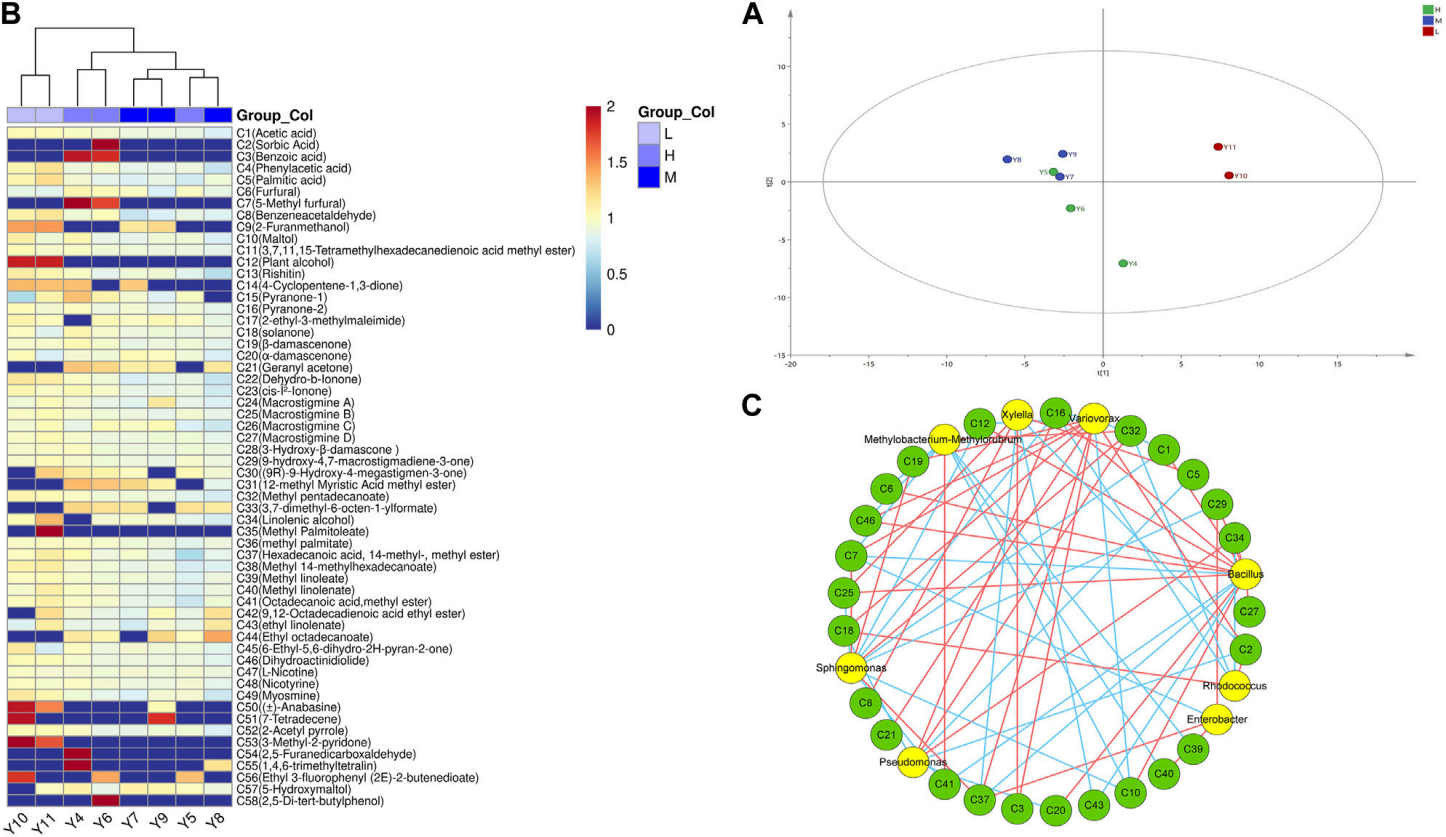
Volatile components in different quality tobacco. **(A)** Principal component analysis; **(B)** Cluster heat map. **(C)** Correlation network analysis diagram of bacterial community and volatile components. L: low grade tobacco; M: medium grade tobacco; H: High grade tobacco.

The volatile aromatic substances of different grades of tobacco were detected using the SPME-GC-MS technique and plotted on PCA and cluster heat maps, as shown in Figures 4A, B. A total of 58 volatile aromatic components were obtained, including 15 ketones, 17 esters, 3 hydrocarbons, 5 acids, 5 aldehydes, and 5 alcohols. Based on the hierarchical clustering of the tobacco samples, it was observed that the volatile substances of high-grade and medium-grade tobacco which had little difference, were clustered into one category and distinguished from low-grade tobacco.

In addition, the correlation between the volatile flavors of tobacco leaves and the surface microbial community was analyzed using the Cytoscape software, and the results were visualized in a network diagram (Figure 4C). *Bacillus* was found to have a higher number of correlated connections, indicating its strong relationship with various flavor substances such as α-damalone (C20), maltol (C10), acetic acid (C1), dihydrokiwifolide (C46), and gigantotrienone D (C27). Furthermore, *Variovorax* showed a positive correlation with 5-hydroxymethylfurfural (C7) and solanone (C18), indicating its relationgship with these flavor substances. It could be inferred that the presence of *Variovorax* contributes to the formation of sweet and scorching aroma in tobacco leaves. *Bacillus* was the dominant bacteria among tobacco leaf microorganisms. It is also the dominant enzyme-producing bacterium reported most frequently. At the same time, the results showed that *Bacillus* was positively correlated with many neutral aroma components in tobacco leaves. Therefore, in this study, we preferentially screened *Bacillus* from the surface of tobacco leaves to study the effect of microbial enzyme production on tobacco quality.

### 3.2 Strain screening and application of enzyme preparations in improving tobacco quality

#### 3.2.1 Strain screening

**FIGURE 5:**
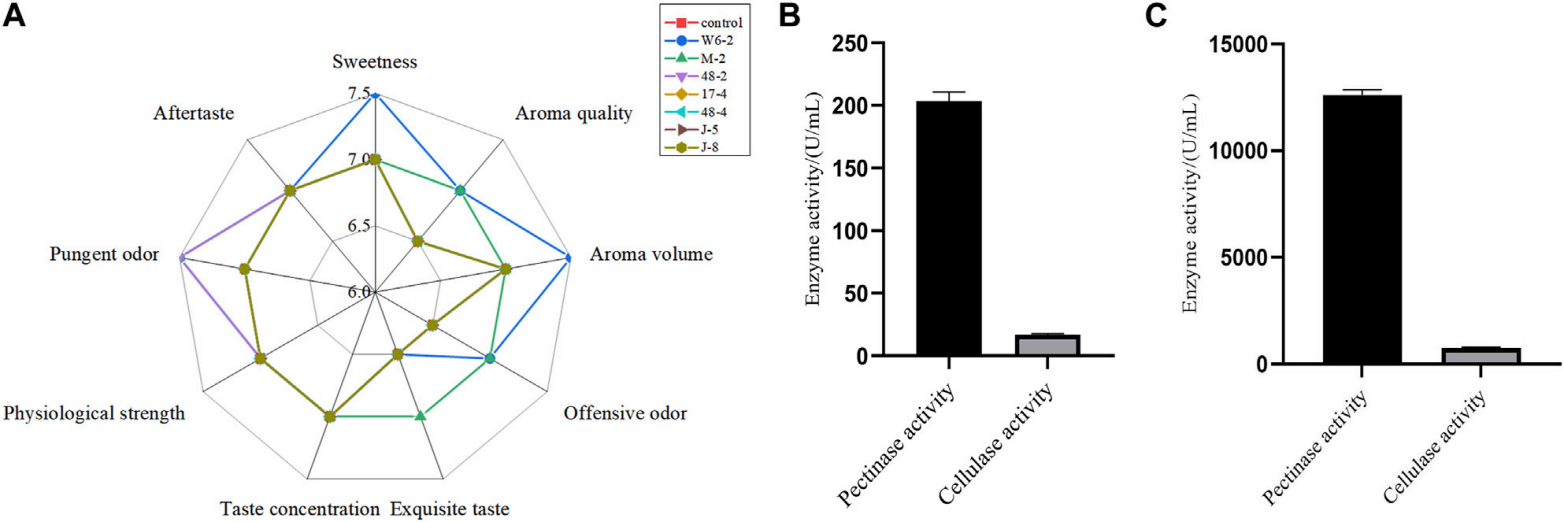
Sensory evaluation and enzyme activity determination. **(A)** Sensory evaluation of tobacco after the effect of the fermentation supernatant of different bacteria on tobacco; **(B)** Enzyme activity determination of fermentation supernatant; **(C)** Determination of enzyme activity in enzyme preparations.

**FIGURE 6:**
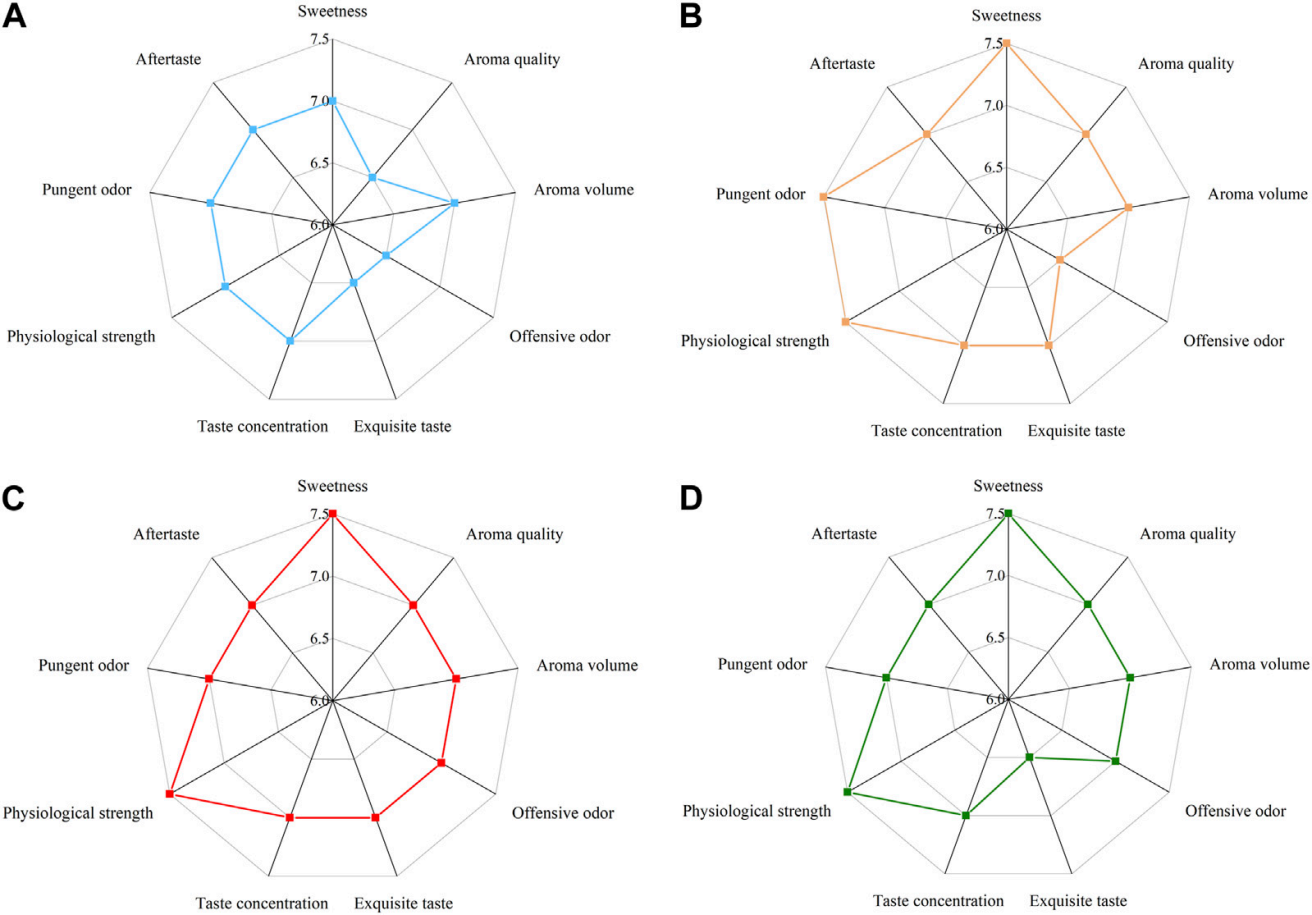
Sensory evaluation radar map of different enzyme additions. **(A)** 0‰; **(B)** 0.2‰; **(C)** 0.3‰ **(D)** 0.5‰.

After sensory evaluation screening (Figure 5A) and determination of pectinase and cellulase activity (Figure 5B), a strain of *Bacillus amylolyticus* W6-2 capable of degrading pectin was screened from the surface of reroasted tobacco leaves in Yunnan Province, and the pectinase activity of the fermentation supernatant reached 203.39 U/mL. The enzyme preparation was obtained through membrane separation, fermentation liquid purification, and ultrafiltration concentration. The pectinase activity reached 1.26 × 10^5^ U/mL and the cellulase activity reached 754 U/mL, as depicted in Figure 5C. The SDS-PAGE experimental analysis of the concentrated liquid (Supplementary Figure S1) showed that the molecular weight of the concentrated liquid was between 30 and 100 kDa. In this experiment, the enzyme preparation was applied to the test tobacco at dosages of 0‰, 0.2‰, 0.3‰, and 0.5‰ mL/g, respectively. The results of sensory evaluation are presented in Figure 6. When the treatment concentration was 0.3‰ mL/g, the flue-cured tobacco demonstrated the best sensory effect. This was characterized by reduced stray gas, decreased strength, increased aroma quantity and sweetness, improved aroma temperament, and smooth smoke.

#### 3.2.2 Effects of enzyme treatment on macromolecules and conventional chemical components of tobacco leaves

**FIGURE 7:**
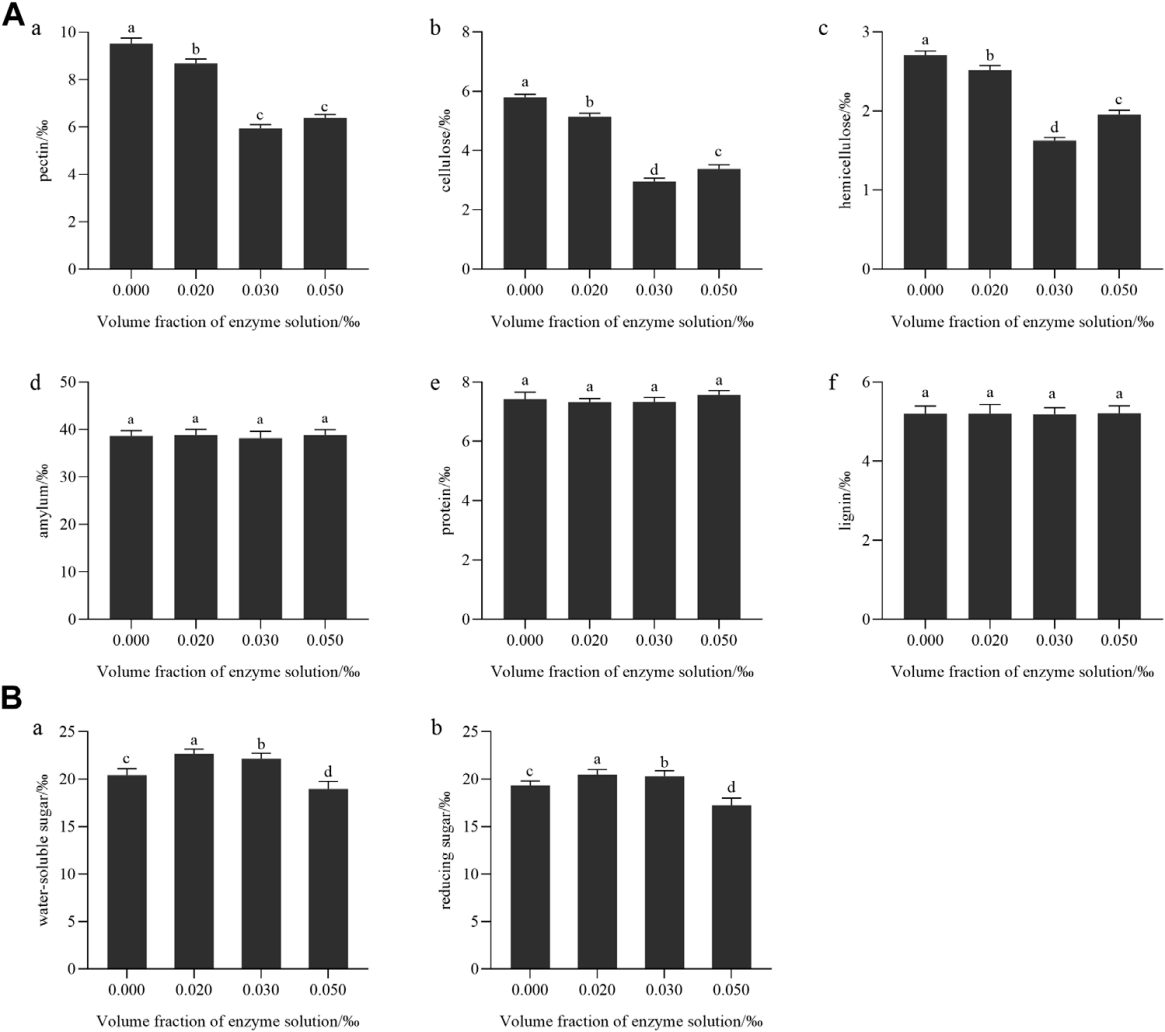
**(A)** Effect of enzyme treatment on the content of macromolecular substances in tobacco leaves: **(a)** pectin; **(b)** cellulose; **(c)** hemicellulose; **(d)** starch; **(e)** protein; **(f)** lignin; **(B)** The effect of enzyme treatment on the content of conventional chemical components: **(a)** Water-soluble total sugar; **(b)** Reducing sugar.

Figure 7A illustrates that when the concentration of enzyme treatment was 0.3‰ (mL/g), the levels of pectin, cellulose, and hemicellulose in tobacco leaves showed the most significant decrease (*p* < 0.05). However, the enzyme treatment had no significant effect on the levels of starch, protein, and lignin in tobacco leaves.

As the enzyme treatment concentration increase in, the content of water-soluble total sugar and reducing sugar in tobacco leaves showed a trend of first increasing and then decreasing, as shown in Figure 7B. It was because when pectinase was added, pectin was hydrolyzed into galacturonic acid and a small amount of monosaccharides (Too et al., 2017), which increased the content of reducing sugar and total sugar in tobacco leaves. However, as the amount of enzyme added increases and the product concentration increases, when the reaction reaches equilibrium, the accumulation of hydrolysis products to a certain concentration would inhibit the hydrolysis of the enzyme, leading to the inhibition of the enzyme’s activity (CAI et al., 2023). Studies showed that total sugar and reducing sugar are positively correlated with aroma quantity, aroma temperament and aftertaste in sensory evaluation indexes (Shi et al., 2013). Combined with the change in macromolecule content after enzyme treatment, it was speculated that 0.2‰–0.3‰ mL/g enzyme solution could transform cellulose, pectin, and other polysaccharides into monosaccharides and oligosaccharides. The chemical composition of tobacco leaves tended to be coordinated, which improved the aroma amount in the smoke, and effectively reduced the irritating and burnt taste of tobacco leaves when smoking, and the sensory effect was the best.

#### 3.2.3 Effects of enzyme treatment on volatile substances

**FIGURE 8:**
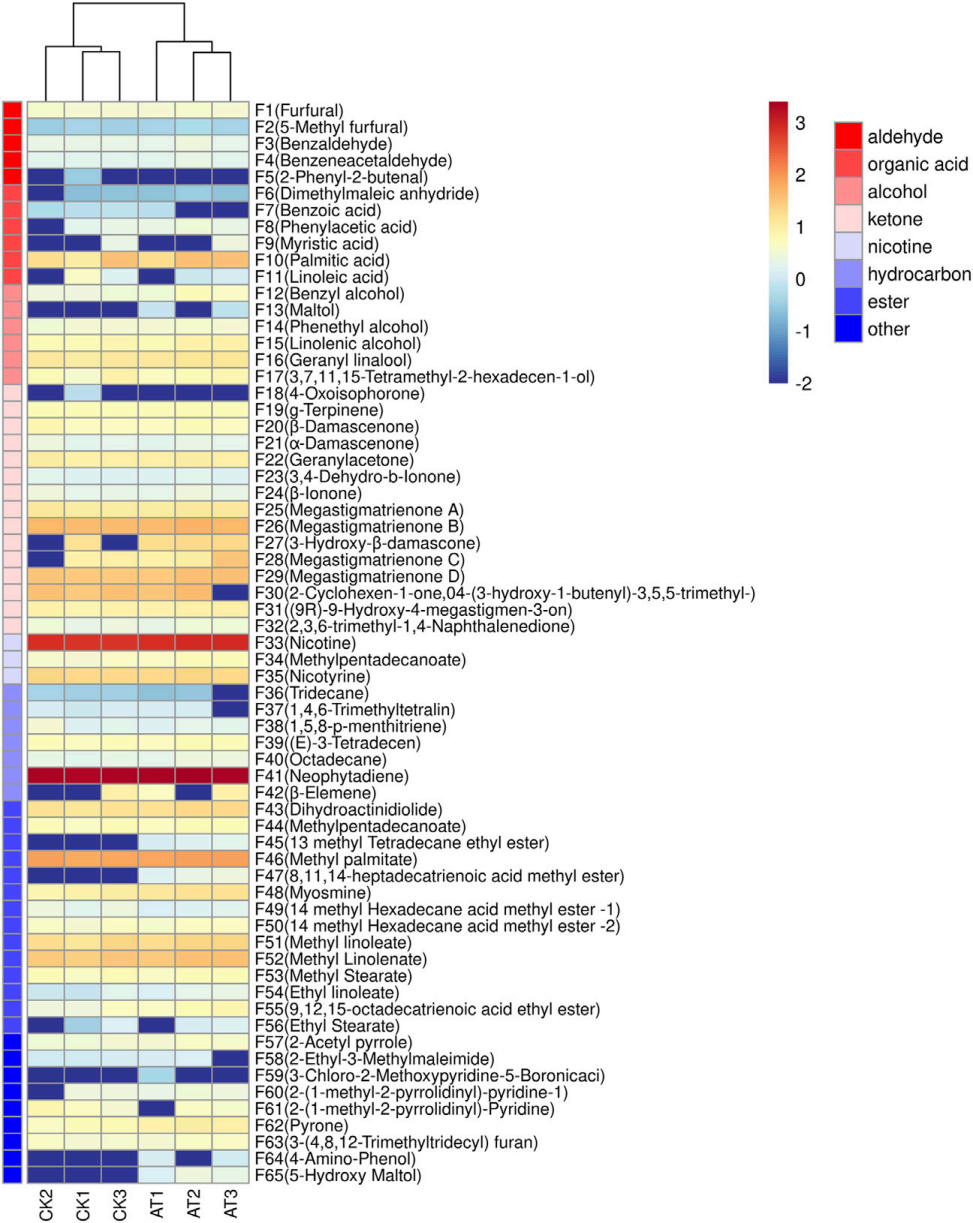
Clustering heat map of volatile components before and after enzyme treatment: CK: control group; AT: enzyme treatment group.

HS-SPME/GC-MS was used to detect the changes of volatile aroma components of flue-cured tobacco treated with 0.3‰ mL/g enzyme preparation. As shown in Figure 8, a total of 63 volatile compounds were identified by GC-MS, including 5 aldehydes, 6 acids, 6 alcohols, 15 ketones and 13 esters. There were no significant differences in the types of volatile compounds between the control group and the group treated with enzyme, but there were significant differences in the content.

**FIGURE 9:**
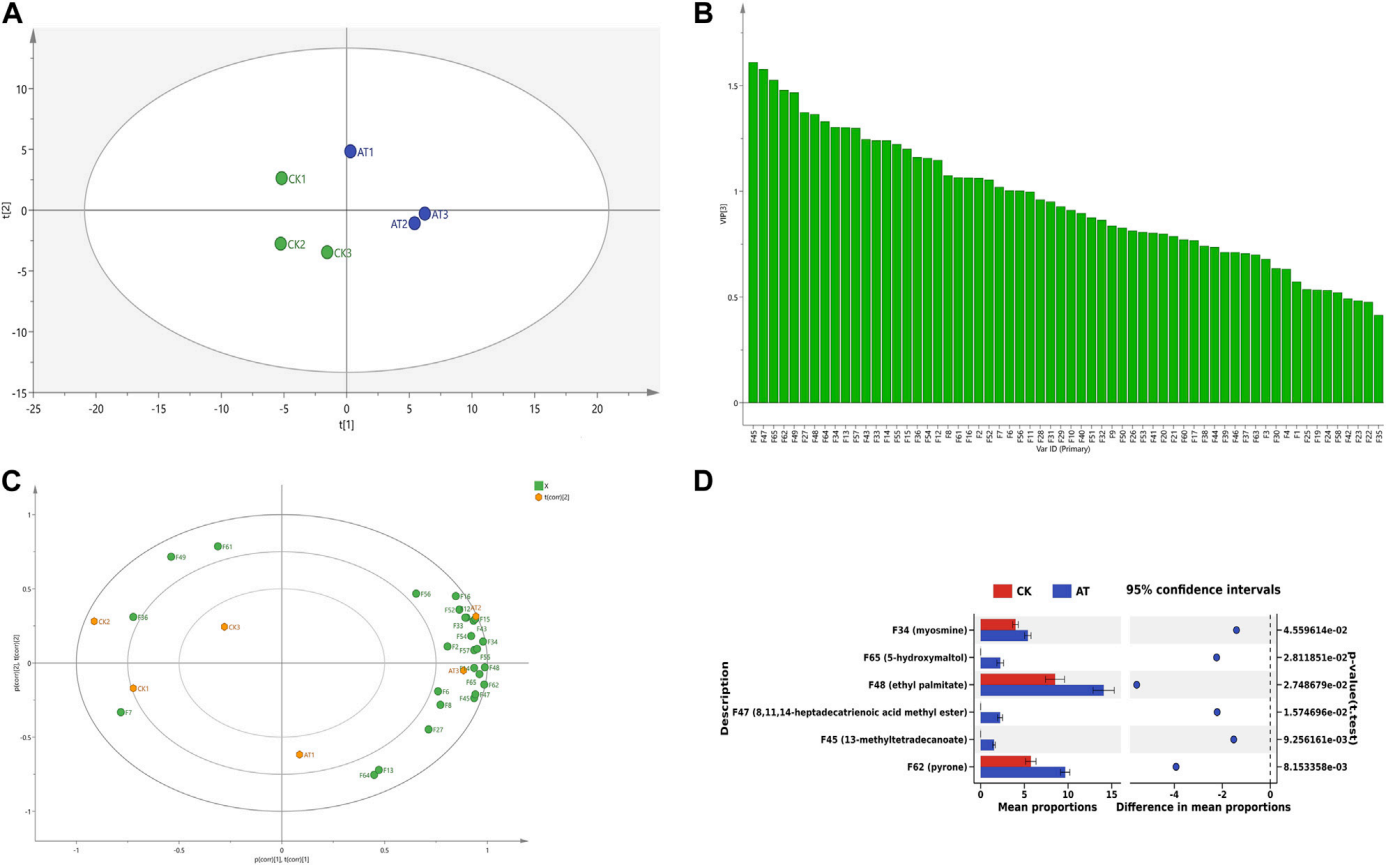
Changes of volatile substances before and after enzyme treatment **(A)** PCA diagram; **(B)** VIP map; **(C)** Load diagram; **(D)** Difference analysis chart: CK: control group; AT: enzyme treatment group.

Principal component analysis (PCA) was used to evaluate the overall changes of volatile components in tobacco leaves before and after enzyme treatment. The results are shown in Figure 9A. The control group was mainly distributed in the second and third quadrants, while the enzyme treatment group was mainly distributed in the first and fourth quadrants. The results showed that volatile substances had obvious regional distribution characteristics before and after enzyme treatment, and had obvious differentiation effects in the PCA diagram.

The Variable importance in the projection (VIP) value can be used to quantify the contribution of each variable to classification (Giannetti et al., 2019). The larger the VIP value, the more significant the difference in volatile components in tobacco leaves before and after enzyme treatment. The VIP values of volatile components in tobacco leaves before and after enzyme treatment were calculated by SIMCA software, as shown in Figure 9B. A total of 27 volatile components were found to have VIP values greater than 1. They are 13-methyltetradecanoate ethyl ester (F45), 5-hydroxymaltol (F65), 4-aminophenol (F64), geranyllinalool (F16), linolenol (F15), 3-hydroxy-β-damalone (F27), ethyl palmitate (F48), dihydrokiwifolide (F43), n-tridecane (F36), maltol (F13), pyranone (F62), etc.

Volatile substances with VIP values greater than 1 were marked on the load diagram to observe the distribution characteristics of volatile components in each region. As shown in Figure 9C, more volatile aroma components were displayed near the position of the enzyme-treated group, indicating that the enzyme-treated tobacco leaves had a greater abundance of aroma substances and more pronounced aroma characteristics. In the control group, the presence of methyl 14-methylhexadecanoate (F49) and benzoic acid (F7) resulted in a grassy and irritating odor (Jiang et al., 2012), and had no positive effect on cigarette smoking. However, in the enzyme treatment group, dihydrokiwifolide (F43), 3-hydroxy-β-dihydrodamalone (F27), and geranyllinalool (F16) provided a fragrant flavor to the tobacco leaves. Carotenoids, which are precursors to many important aroma components, played a crucial role in the formation of tobacco aroma quality (Popova et al., 2019), with their degradation product being 3-hydroxy-β-dihydrodamalone. This compound possesses a soft, sweet, and floral aroma, contributing to a mellow and delicate tobacco aroma. In summary, the enzyme treatment improved the aroma quality of tobacco leaves, resulting in a similar aroma profile to medium and fine tobacco, characterized by fresh, burnt, and sweet aromas accompanied by subtle floral and clear sweet aromas.

The changes in aroma characteristics and content of volatile components in tobacco leaves were one of the reasons for the improvement of sensory effects of tobacco leaves, and there were many types of substances that represent the aroma characteristics of tobacco leaves when treated with enzymes. In order to further clarify the changes in the content of specific volatile flavor substances, a differential analysis of important volatile components (VIP >1) was carried out, and the results are shown in Figure 9D. The contents of pyranone (F62) and ethyl palmitate (F48) increased significantly after enzyme treatment, and 5-hydroxymaltol (F65), 13-methyltetradecanoate (F45), and methyl 8,11,14-heptadecatrienoate (F47) were produced. Ester compounds are very important flavor substances, that can eliminate the irritating effect of tobacco leaves, and increase the concentration of tobacco aroma, giving the smoke a mellow taste. Pyranone has a roasted and caramel flavor (Salmeron et al., 2014). 5-hydroxymaltol is produced by sugar pyrolysis, and has a burnt flavor.

OAV values can provide insights into the contribution of aroma components to the overall aroma, considering their concentration and threshold values (Patton and Josephson, 1957). In the analysis of food flavor, aroma components with OAV values greater than 1 are generally considered to contribute to the overall aroma, with a higher value indicating a higher contribution. Table 2 presents the OAV analysis results of the selected important volatile components (VIP >1). Benzyl alcohol, ethyl palmitate, phenylethanol, dihydrokiwifolide, and other compounds had the highest OAV values, indicating their significant contribution to the overall aroma.

**TABLE 2:** Aroma activity value of each volatile substance.

No.	Characteristic components	Before enzyme treatment (μg/g)	After enzyme treatment (μg/g)	Threshold (μg/g)	OAV
F2	5-Methylfuranal	0.36 ± 0.03	0.44 ± 0.06	0.25	1.76 ± 0.26
F13	Maltol	0.00	0.81 ± 0.01	0.479	1.69 ± 0.02
F14	Phenethyl alcohol	3.26 ± 0.21	3.61 ± 0.13	0.56	6.44 ± 0.23
F43	Dihydrokiwifolide	15.19 ± 1.25	18.32 ± 1.17	3.8	4.82 ± 0.47
F48	Ethyl palmitate	8.51 ± 0.99	14.07 ± 1.15	2	7.04 ± 0.58
F54	Ethyl linoleate	1.18 ± 0.49	1.99 ± 0.51	0.45	4.42 ± 1.14
F57	2-Acetyl pyrrole	3.16 ± 0.23	3.85 ± 0.35	59	0.07 ± 0.01
F12	Benzyl alcohol	2.82 ± 0.14	4.28 ± 0.70	0.1	42.76 ± 6.96

These volatile substances with high OAV values are essentially the primary odorant substances in tobacco leaves. Benzyl alcohol, which produced by the conversion of phenylalanine, is an important odorant in cigarettes (Li et al., 2017). Dihydrokiwifolide can bring fragrance to tobacco leaves and has the effect of softening smoke. Phenethyl alcohol has a floral scent, and 2-acetyl pyrrole has a bread-like, toasty aroma (Xiao et al., 2015). The enzyme treatment promoted the formation of tobacco aroma.

By comparing the volatile compounds that changed significantly before and after the enzyme treatment, the test results showed that the enzyme treatment was beneficial for increasing the content of esters, ketones and aldehydes in tobacco and reducing benzoic acid, which has reduced the grassy impurity and irritability of flue-cured tobacco, and had a significant effect on enhancing aroma and removing pungent odor.

## 4 Discussion

In order to explore the effects of microorganisms on tobacco leaves, high-throughput sequencing was used to analyze the composition and diversity of microbial communities in tobacco samples of different grades in Yunnan Province, and PICRUSt was used to predict the microbial functions on the surface of tobacco leaves. Additionally, PICRUSt was utilized to predict the microbial functions on the surface of tobacco leaves. Previous research has indicated the importance of microbial communities and their activities in improving tobacco leaves quality. For instance, Li et al. (2020) found that microorganisms, such as *Pseudomonas* and *Sphingosinomonas*, as well as metabolites, like plant sphingosine and sphingosine, play a crucial role in tobacco fermentation. Metabolites influenced the quality of cigarettes through the sphingosine metabolism pathway. According to the high-throughput sequencing results, *Phagocyta*, *Sphingosinomonas*, *Pseudomonas*, and *Bacillus* were the dominant bacteria on the surface of Yunnan tobacco leaves. These bacteria participate in the aging process of flue-cured tobacco through metabolic processes, with amino acid metabolism and carbohydrate metabolism being the main pathways for bacterial degradation of macromolecular substances. Carbon metabolism and nitrogen metabolism are two major metabolic systems involved in tobacco leaf formation, which are related to the components that mainly affect the quality of tobacco (Ma et al., 2024). Ning et al. (2023) found that after tobacco leaves were treated with amylase, at the genus level, the dominate bacterial community of tobacco fermentation was mainly composed of *Bacillus*, *Sphingomonas*, *Pseudomonas*, *Methylobacterium*, *Acinetobacter*, *Pantoea*. Among the metabolic pathways, the metabolic abundance of carbohydrate metabolism, amino acid metabolism and energy metabolism is relatively high. And the carbohydrate metabolism was mainly composed of pyruvate metabolism, glyoxylate and dicarboxylate metabolism, and glycolysis/gluconeogenesis.

Based on the results of the high-throughput prediction, *Bacillus amyloliquefaciens* W6-2, which could produce pectinase, was isolated from the surface of tobacco. After the fermentation liquid was separated, purified by membrane, concentrated by ultrafiltration, pectinase preparations with pectinase activity reaching 1.26 × 10^5^ U/mL and cellulase activity reaching 754 U/mL were prepared and applied to tobacco leaves at a treatment concentration of 0.3‰ mL/g. It was found that the contents of macromolecular substances such as pectin, cellulose, and hemicellulose decreased significantly after enzyme treatment, while the contents of water-soluble total sugar and reducing sugar increased. The pectinase produced by W6-2 broke the connection of macromolecular substances in the cell wall skeleton structure of tobacco leaves, effectively reduced the content of cell wall substances in tobacco leaves, degraded pectin into small molecular reducing sugar, increased the reducing sugar content, reduced the irritation of tobacco leaves, and improved the sweetness of tobacco leaves (Huang et al., 2015). The accumulation of reducing sugars contributes to the formation of Maillard reaction products, enhancing aroma and flavor, thus providing nutty, sweet, or popcorn flavors, which helps improve the softness and aromatic strength of flue-cured tobacco (Michael et al., 2018). The degradation of cellulose and hemicellulose can reduce the irritability of tobacco smoke (Kaelin et al., 1994). In this study, through sensory evaluation, it was found that after enzyme treatment, the impurities and irritability of flue-cured tobacco decreased, while the aroma, sweetness, and fineness increased. The results indicated that the treatment with enzyme preparation could improve the quality of flue-cured tobacco by degrading macromolecules.

Geranyllinalol, dihydrokiwifolide, and 3-hydroxy-β-dihydrodamalone were found in higher concentrations in the enzyme-treated group, and these compounds are the degradation products of carotenoids. In tobacco, terpenoids and their degradation products play a crucial role in the generation aroma, especially the degradation products of carotenoids (Lewinsohn et al., 2005). These compounds are the main aromatic constituents that have strong fruity, woody, and violet aromas, which enhance the sensory experience of tobacco smoke. The carotenoid degradation pathway is considered as a key pathway in the formation of aromatic compounds in various plants and plant-based products. Carotenoids undergo enzymatic or chemical reactions that break double bonds at different positions, ultimately producing various C9-C13 norisoprenoid aroma components. Geranyllinalools, which are monoterpenoids with a tetrahydrofuran structure, are sweet and earthy compounds with floral and fruity flavors (Gong et al., 2021). Moreover, the content of volatile substanced such as furfural, 5-methylfurfural, and pyranones increased after enzyme treatment. It is speculated that, under specific conditions, a series of Maillard reaction products are formed through a reaction between reducing sugars and amino acids. Furfural and its derivatives undergo an Amadori rearrangement, leading to the synthesis of reduced ketone heterocyclic compounds, such as pyrazines, furans, and pyrroles (Gong et al., 2021). The degradation of phenylalanine results in an increased phenylethanol content, which imparts a fresh rose aroma (Gaoming et al., 2015). Thus, it can be inferred that carotenoid degradation and Maillard reactions occured in flue-cured tobacco after enzyme treatment, leading to an increase in the content of reaction products. These substances contributed to the fruity and caramel flavors of flue-cured tobacco. Li-Yuan Zhang et al. (Zhang et al., 2024) screened *Bacillus velezensis* A2 and *Bacillus endophyticus* A4 from tobacco leaf. After co-fermentation of the two strains, the aroma components of neophydiene, solanone and dihydrokiwifolide in tobacco leaf significantly increased. Therefore, in this study, there may be a potential relationship between the production of these aroma substances and the carotenoid degradation of tobacco leaves by W6-2.

The formation of new esters after enzyme treatment, such as 13-methyltetradecanoate and methyl 8,11,14-heptadecatrienoate, contributed to the sweet taste. Esters are crucial flavor components and are generally formed through the esterification reaction between alcohols produced from the oxidative degradation of fatty acids and small-molecule free fatty acids. Esters can reduce the irritation of tobacco leaves and enhance the aroma concentration, resulting in a smoother taste of smoke (Buntić et al., 2019). Through OAV analysis, it has been determined that ethyl palmitate, dihydrokiwifolide, and ethyl linoleate were three ester compounds with high OAV values. Among them, ethyl palmitate not only showed a significant difference but also had a high OAV value. Lipids in tobacco leaves can be hydrolyzed by lipases to produce ethyl palmitate (Saerens et al., 2008). Ethyl palmitate is an ester compound that imparts alcohol and smoke-like flavors, as well as a hint of vanilla. Thereby, it increases the concentration of tobacco aroma. It is speculated that the observed significant increase in the content of ester compounds, particularly ethyl palmitate, can be attributed to the presence of a small amount of lipase in the enzyme preparation or the condensation reaction between alcohol and acid in tobacco leaves. This increase in ester substances contributes to the softening of the smoke and enhances the smoothness of smoking.

Methyl 14-methylhexadecanoate and benzoic acid were decreased following enzyme treatment. Benzoic acid is known for its sour taste and spicy flavor, and its strong odor can cause irritation, often leading to cough when inhaled (Otero-Losada, 1999). This is undesirable for smoking flue-cured tobacco. The decrease in these two compounds, along with the degradation of cellulose and hemicellulose, contributes to a decrease in the odor of flue-cured tobacco. Therefore, applying pectinase preparations to tobacco leaves is considered an effective method for enhaning the quality of tobacco.

## 5 Conclusion

The microbial resources present on tobacco leaves play a crucial role in influencing the quality of tobacco. In this study, the microbial community diversity of in Yunnan flue-cured tobacco were analyzed using high-throughput sequencing techniques. The results revealed that the variation in flavor between the high and medium grades of flue-cured tobacco and the lower grade could be attributed to differences in dominant bacteria groups. There were more bacteria that could degrade macromolecules, such as *Bacillus*, in high and medium grades of tobacco, which was positively correlated with key odorant components in tobacco leaves. The strain of W6-2, which can produce pectinase on the surface of Yunnan tobacco leaves, was further screened out. The enzyme preparation of W6-2 reduced the contents of pectin and cellulose decreased, and the contents of carotenoid degradation products, Maillard reaction products and ester substances increased, which brought fruity flavor, sweetness and delicacy to flue-cured tobacco. The decrease of benzoic acid content alleviated the irritation and green sensation of flue-cured tobacco. Through the use of high-throughput sequencing and predictive analysis, this study aimed to purposefully screen functional microorganisms that can degrade macromolecular substances in order to enhance the sensory properties of tobacco leaves. The findings from this study provide a foundation for the development of artificial fermentation methods to improve the overall quality of tobacco.

## Data Availability

The original contributions presented in the study are publicly available. This data can be found here: https://www.ncbi.nlm.nih.gov/sra/?term=PRJNA1058426.

## References

[B1] Ben-Arie R. Sonego L. (1980). Pectolytic enzyme activity involved in woolly breakdown of stored peaches. Phytochemistry 19 (12), 2553–2555. 10.1016/s0031-9422(00)83917-5

[B2] Buntić A. Milić M. Stajković-Srbinović O. S. Rasulić N. V. Delić D. I. Mihajlovski K. (2019). Cellulase production by Sinorhizobium meliloti strain 224 using waste tobacco as substrate: utilization of waste tobacco for cellulase production. Int. J. Environ. Sci. Technol. (Tehran) 16, 5881–5890. 10.1007/s13762-019-02230-9

[B3] Cai W. Zhang Q. Pengcheng Z. Wanrong H. Yun J. Yonghong Y. (2023). High throughput screening of key functional strains based on improving tobacco quality and mixed fermentation. Front. Bioeng. Biotech. 11, 3. 10.3389/fbioe.2023.1108766 PMC9880406 36714011

[B4] Cheng T. H. Ismail N. Kamaruding N. Saidin J. Danish-Daniel M. (2020). Industrial enzymes-producing marine bacteria from marine resources. Biotechnol. Rep. 27, e00482. 10.1016/j.btre.2020.e00482 PMC7267704 32514406

[B5] Dai X. H. He J. Yan H. Li N. Dai L. L. Dong B. (2017). Effects of free ammonia regulation on the performance of high solid anaerobic digesters with dewatered sludge. Huan jing ke xue 38 (2), 679–687. 10.13227/j.hjkx.201607137 29964526

[B6] Dai Y. Y. Zhu L. Liu S. Yu H. (2013). Analytical method of free and conjugated neutral aroma components in tobacco by solvent extraction coupled with comprehensive two-dimensional gas chromatography–time-of-flight mass spectrometry. J. Chromatogr. A 1280, 122–127. 10.1016/j.chroma.2013.01.028 23357748

[B7] Deng G. Li X. Li C. Zhou J. Zhang Z. (2003). Study on the improvement of tobacco leaf quality by pectin reducing bacteria. Tob. Sci. Technol. 11, 3. 10.3969/j.issn.1002-0861.2003.11.004

[B8] Dorokhov Y. L. Shindyapina A. V. Sheshukova E. V. Komarova T. V. (2015). Metabolic methanol: molecular pathways and physiological roles. Physiol. Rev. 95 (2), 603–644. 10.1152/physrev.00034.2014 25834233

[B9] English C. F. Bell E. J. Berger A. J. (1967). Isolation of thermophiles from broadleaf tobacco and effect of pure culture inoculation on cigar aroma and mildness. Appl. Microbiol. 15 (1), 117–119. 10.1128/am.15.1.117-119.1967 16349710 PMC546854

[B10] Gaoming R. P. A. Lei L. Xuesong L. Zhang A. (2015). Fast quantification of phenylethyl alcohol in rose water and chemical profiles of rose water and oil of rosa damascena and rosa rugosa from southeast China. J. Liq. Chromatogr. Relat. Technol. 38 (7), 823–832. 10.1080/10826076.2014.976710

[B11] Giannetti V. Mariani M. B. Torrelli P. Marini F. (2019). Flavour component analysis by HS-SPME/GC–MS and chemometric modeling to characterize Pilsner-style Lager craft beers. Microchem. J. 149, 103991. 10.1016/j.microc.2019.103991

[B12] Gong M. Zhou Z. Liu S. Zhu S. Mao J. Zhong F. (2021). Formation pathways and precursors of furfural during zhenjiang aromatic vinegar production. Food Chem. 354 (2), 129503. 10.1016/j.foodchem.2021.129503 33743446

[B13] Hu B. Gu K. Gong J. Zhang K. Chen D. He X. (2021). The effect of flue-curing procedure on the dynamic change of microbial diversity of tobaccos. Sci. Rep. 11 (1), 5354. 10.1038/s41598-021-84875-6 33686144 PMC7940495

[B14] Huang T. Gui J. Zheng L. (2015). Research progress on improving quality of reconstituted tobacco leaf with enzyme preparation. J. Northeast Agric. Univ. 46 (10), 7. 10.19720/j.cnki.issn.1005-9369.2015.10.016

[B15] Jiang H. zhao M. Liu P. Zhai X. (2012). Research progress on flavor classification and quality characteristics of flue-cured tobacco. J. Zhejiang Agric. Sci. (12), 1628–1632. 10.3969/j.issn.0528-9017.2012.12.007

[B16] Kaelin P. Gadani F. (2000). Occurrence of Bacillus thuringiensis on cured tobacco leaves. Curr. Microbiol. 40 (3), 205–209. 10.1007/s002849910041 10679055

[B17] Kaelin P. Morel P. Gadani F. (1994). Isolation of Bacillus thuringiensis from stored tobacco and lasioderma serricorne (F.). Appl. Environ. Microbiol. 60 (1), 19–25. 10.1128/aem.60.1.19-25.1994 16349149 PMC201263

[B18] Lewinsohn E. Sitrit Y. Bar E. Azulay Y. Ibdah M. Meir A. (2005). Not just colors—carotenoid degradation as a link between pigmentation and aroma in tomato and watermelon fruit. Trends Food Sci. Technol. 16 (9), 407–415. 10.1016/j.tifs.2005.04.004

[B19] Li J. He J. Shi H. Li Z. Peng X. Chen L. (2017). Study on characteristic aroma components of Guangyuan flue-cured tobacco leaves. J. Anhui Agric. Sci. 45 (22), 65–68. 10.13989/j.cnki.0517-6611.2017.22.020

[B20] Li J. Zhao Y. Qin Y. Shi H. (2020). Influence of microbiota and metabolites on the quality of tobacco during fermentation. BMC Microbiol. 20 (1), 356–415. 10.1186/s12866-020-02035-8 33213368 PMC7678276

[B21] Longjie Z. Hua Z. Yang W. Jingbo C. Yi C. Huiyun L. (2020). Types and contents of key aroma components produced by alkalineMaillard reaction. Tob. Sci. Technol. 53 (6). 10.16135/j.issn1002-0861.2019.0395

[B22] Ma L. Wang Y. Wang X. Lü X. (2024). Solid-state fermentation improves tobacco leaves quality via the screened Bacillus subtilis of simultaneously degrading starch and protein ability. Appl. Biochem. Biotechnol. 196 (1), 506–521. 10.1007/s12010-023-04486-x 37148443

[B23] Man Z. Jingli W. Tao W. Gao L. Yin H. Lü X. (2017). The purification and characterization of a novel alkali-stable pectate lyase produced by Bacillus subtilis PB1. World J. Microbiol. Biotechnol. 33 (10), 190. 10.1007/s11274-017-2357-8 28975516

[B24] Michael H. Evangeline S. Davy V. Ann D. W. Amelia T. Leonardo S. (2018). Assessing the influence of pod storage on sugar and free amino acid profiles and the implications on some Maillard reaction related flavor volatiles in Forastero cocoa beans. Food Res. Int. 111, 607–620. 10.1016/j.foodres.2018.05.064 30007725

[B25] Ning Y. Mai J. Hu B.-B. Lin Z.-L. Chen Y. Jiang Y.-L. (2023). Study on the effect of enzymatic treatment of tobacco on HnB cigarettes and microbial succession during fermentation. Appl. Microbiol. Biotechnol. 1, 4217–4232. 10.1007/s00253-023-12577-2 37209161

[B26] Otero-Losada M. E. (1999). A kinetic study on benzoic acid pungency and sensory attributes of benzoic acid. Chem. Senses 24 (3), 245–253. 10.1093/chemse/24.3.245 10400442

[B27] Pan Y. Zheng X. Xiang Y. (2021). Structure-function elucidation of a microbial consortium in degrading rice straw and producing acetic and butyric acids via metagenome combining 16S rDNA sequencing. Bioresour. Technol. 340, 125709. 10.1016/j.biortech.2021.125709 34375790

[B28] Patton S. Josephson D. V. (1957). A METHOD FOR DETERMINING SIGNIFICANCE OF VOLATILE FLAVOR COMPOUNDS IN FOODS^a^ . Food Res. 22 (3), 316–318. 10.1111/j.1365-2621.1957.tb17017.x

[B29] Popova V. Ivanova T. Prokopov T. Nikolova M. Zheljazkov V. D. (2019). Carotenoid-related volatile compounds of tobacco (nicotiana tabacum L.) essential oils. Molecules 24 (19), 3446. 10.3390/molecules24193446 31547525 PMC6804150

[B30] Reid J. McKinstry D. Haley D. (1937). The fermentation of cigar-leaf tobacco. Science 86 (2235), 404. 10.1126/science.86.2235.404-a 17832645

[B31] Saerens S. Delvaux F. Verstrepen K. Van Dijck P. Thevelein J. Delvaux F. (2008). Parameters affecting ethyl ester production by *Saccharomyces cerevisiae* during fermentation. Appl. Environ. Microbiol. 74 (2), 454–461. 10.1128/AEM.01616-07 17993562 PMC2223249

[B32] Salmeron I. Rozada R. Thomas K. Ortega-Rivas E. Pandiella S. S. (2014). Sensory characteristics and volatile composition of a cereal beverage fermented with Bifidobacterium breve NCIMB 702257. Food Sci. Technol. Int. 20 (3), 205–213. 10.1177/1082013213481466 23744118

[B33] Shen D. Gu S. (2009). The mechanism for thermal decomposition of cellulose and its main products. Bioresour. Technol. 100 (24), 6496–6504. 10.1016/j.biortech.2009.06.095 19625184

[B34] Shi F. X. Wang H. Y. Zhang T. Sun L. Mou D. R. Zou Q. (2013). Correlation between smoking quality and smoke components,physical index and chemical components of cigarette. J. South. Agric., 486–492. 10.3969/j:issn.2095-1191.2013.3.486

[B35] Shiratori H. Sasaya K. Ohiwa H. Ikeno H. Ayame S. Kataoka N. (2009). Clostridium clariflavum sp. nov. and Clostridium caenicola sp. nov., moderately thermophilic, cellulose-/cellobiose-digesting bacteria isolated from methanogenic sludge. Int. J. Syst. Evol. Microbiol. 59 (7), 1764–1770. 10.1099/ijs.0.003483-0 19542130

[B36] Tao J. Chen Q. Chen S. Lu P. Chen Y. Jin J. (2022). Metagenomic insight into the microbial degradation of organic compounds in fermented plant leaves. Environ. Res. 214, 113902. 10.1016/j.envres.2022.113902 35839908

[B37] Too S. Chua K. O. Lim Y. L. Chen J. W. Convey P. Mohidin T. B. M. (2017). Complete genome sequence of Planococcus donghaensis JH1 T, a pectin-degrading bacterium. J. Biotechnol. 252, 11–14. 10.1016/j.jbiotec.2017.05.005 28483443

[B38] Torikaiu K. Uwano Y. Nakamori T. Tarora W. Takahashi H. (2005). Study on tobacco components involved in the pyrolytic generation of selected smoke constituents. Food Chem. Toxicol. 43 (4), 559–568. 10.1016/j.fct.2004.12.011 15721203

[B39] Wang J. Xu Z. Fan J. Wang Y. Tian Z. Chen Y. (2015). Effects of X-ray irradiation on the microbial growth and quality of flue-cured tobacco during aging. Radiat. Phys. Chem. 111, 9–13. 10.1016/j.radphyschem.2015.02.005

[B40] Wang L. F. Lee J. Y. Chung J. O. Baik J. H. So S. Park S. K. (2008). Discrimination of teas with different degrees of fermentation by SPME-GC analysis of the characteristic volatile flavour compounds. Food Chem. 109 (1), 196–206. 10.1016/j.foodchem.2007.12.054 26054281

[B41] Wang Z. Y. Shao Y. Zhou Q. M. Chen G. Juan L. I. (2013). The pectin and its influence on tobacco physiology and quality. J. Agric. Sci. Technol. 10.3969/j.issn.1008-0864

[B42] Wu X. Zhu P. Li D. Zheng T. Cai W. Li J. (2021). Bioaugmentation of Bacillus amyloliquefaciens–Bacillus kochii co-cultivation to improve sensory quality of flue-cured tobacco. Arch. Microbiol. 203, 5723–5733. 10.1007/s00203-021-02556-4 34480626

[B43] Wu Z. Weeks W. W. Long R. C. (1992). Contribution of neutral volatiles to flavor intensity of tobacco during smoking. J. Agric. Food. Chem. 40 (10), 1917–1921. 10.1021/jf00022a038

[B44] Xiao Z. Wu M. Niu Y. Chen F. Zhang X. Zhu J. (2015). Contribution of chicken base addition to aroma characteristics of Maillard reaction products based on gas chromatography-mass spectrometry, electronic nose, and statistical analysis. Food Sci. Biotechnol. 24 (2), 411–419. 10.1007/s10068-015-0054-7

[B45] Yu J. Ma H. Yang H. Dong G. (2009). Study on the degradation of pectin in tobacco leaf by pectinase. Acta Agric. Jiangxi 21 (03), 136–138. 10.19386/j.cnki.jxnyxb.2009.03.044

[B46] Zhang L.-Y. Mai J. Shi J.-F. Ai K.-B. He L. Zhu M.-J. (2024). Study on tobacco quality improvement and bacterial community succession during microbial co-fermentation. Ind. Crops Prod. 208, 117889. 10.1016/j.indcrop.2023.117889

[B47] Zhou J. Yu L. Zhang J. Zhang X. Xue Y. Liu J. (2020). Characterization of the core microbiome in tobacco leaves during aging. Microbiologyopen 9 (3), e984. 10.1002/mbo3.984 31893578 PMC7066457

