# Supplementary figures and images for: Application of pectin hydrolyzing bacteria in tobacco to improve flue-cured tobacco quality
Source: Front Bioeng Biotechnol. 2024 Mar 7;12:1340160. doi: 10.3389/fbioe.2024.1340160 (PMC10955059; doi:10.3389/fbioe.2024.1340160)

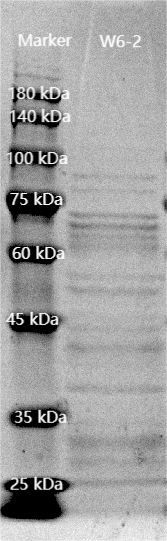


FIGURE S1 SDS-PAGE analysis of W6-2 concentrated liquid

Supplement: Supplementary file 1 [file Table1.DOCX]
